# Predicting natural conception leading to live birth for couples with infertility: a single-centre population-based cohort study of 7086 couples

**DOI:** 10.1093/hropen/hoag056

**Published:** 2026-06-13

**Authors:** Natalie J Cameron, Kate Brian, David J McLernon, Siladitya Bhattacharya

**Affiliations:** Aberdeen Centre for Women’s Health Research, School of Medicine, Medical Sciences and Nutrition, University of Aberdeen, Aberdeen, UK; The Fertility Alliance, London, UK; Biostatistics and Health Data Science, University of Aberdeen, Aberdeen, UK; School of Medicine, Medical Sciences & Nutrition, University of Aberdeen, Aberdeen, UK

**Keywords:** infertility, IVF, live birth, prediction model, natural conception

## Abstract

**STUDY QUESTION:**

Can a model be developed to predict natural conception leading to live birth over a period of 12 months in couples with different causes of infertility?

**SUMMARY ANSWER:**

We developed and validated a novel clinical prediction model that can be used to provide individualized estimates of the chance of a natural conception (leading to live birth) over a 12-month period for couples with different causes of infertility, including tubal factor, anovulation, male factor, unexplained, other (cervical, uterine, or sexual factor), and endometriosis.

**WHAT IS KNOWN ALREADY:**

Existing prediction models for natural conception are primarily aimed at infertile couples with no identified cause (unexplained), mild male factor, or minimal endometriosis. Currently, there is no available model that is applicable across a wide range of fertility diagnoses at the point of initial assessment.

**STUDY DESIGN, SIZE, DURATION:**

A population-based cohort study based on data from a single large tertiary UK fertility centre serving the entirety of the North of Scotland, including all 9757 couples who registered at the clinic for the first time from 1998 to 2015.

**PARTICIPANTS/MATERIALS, SETTING, METHODS:**

Using a Cox proportional hazards survival model, we estimated the chance of conception within the first year from diagnosis of infertility leading to live birth. The predictive accuracy of the model was assessed using discrimination and calibration measures in an internal validation. The clinical utility of the model was assessed using decision curve analysis.

**MAIN RESULTS AND THE ROLE OF CHANCE:**

After exclusions, 7086 couples with infertility were included, of whom 891 (13%) had a natural conception within 1 year of diagnosis leading to live birth. Longer duration of infertility [hazard ratio (HR): 0.64 (95% CI: 0.57–0.72)], increasing female age [0.62 (0.55–0.70)], and tubal infertility [0.62 (0.47–0.81)] had the strongest influence on reducing the chance of natural conception within 1 year leading to live birth. Conversely, previous pregnancy [1.30 (1.12–1.50)] and unexplained infertility [1.29 (1.00–1.67)] were most strongly associated with increased chances of live birth. For example, a 25-year-old woman with 1 year of secondary unexplained infertility has a 33.8% predicted chance of having a baby resulting from natural conception within a year of diagnosis. In contrast, a 35-year-old woman with tubal infertility and 1 year of primary infertility has an estimated 6.0% predicted chance of live birth over a similar time horizon. The model demonstrated acceptable discrimination (optimism-corrected C-statistic 0.645) and good calibration in internal validation. Decision curve analysis demonstrated that applying the model at a 20% probability threshold would correctly identify an additional 7.2 couples per 100 as being likely to benefit from fertility treatment when compared against a ‘treat-all’ policy, which incorrectly assumes that all couples would benefit from fertility treatment. A freely available online calculator was constructed from the model formula to generate individual estimates of the chance of live birth following natural conception.

**LIMITATIONS, REASONS FOR CAUTION:**

The data used to inform this model were collected from a single centre until 2016, limiting generalizability. Any information not collected in this dataset, such as markers of ovarian reserve, severity of endometriosis, or subtype of ovulatory disorder, could not be included and may have improved the accuracy of the model predictions.

**WIDER IMPLICATIONS OF THE FINDINGS:**

As we have demonstrated that couples diagnosed with infertility can conceive on their own, knowledge of their individual chances of having a baby can clarify the net value of active treatment, including assisted reproduction. Use of this model via the online calculator could improve the quality of decision-making around the nature and timing of initiating fertility treatment.

**FUNDING:**

This work was funded in part by an NHS Grampian Charities Small Research Grant awarded to N.J.C, grant number SRG 24-30. We acknowledge the associated financial support of NHS Research Scotland, through NHS Grampian investment in the Grampian DaSH.

**DISCLOSURES:**

N.J.C has no conflict of interest to declare. K.B. declares a previous role as Founder of the Fertility Alliance (charity) and receipt of speaker fees/honoraria from Merck and IBSA (paid to the Fertility Alliance). D.J.M. reports consulting fees from the Society for Assisted Reproductive Technology (SART) (paid to institution), honoraria from Merck for a fertility prediction modelling workshop, and financial support from ESHRE, Merck and IVIRMA to present at scientific conferences. S.B. reports royalties/licenses from Cambridge University Press, consulting fees from Merck, Ferring and Organon (paid to S.B. and to the institution), speaker fees/honoraria from Merck (paid to S.B. and to the institution), support for attending meetings/travel from Merck, and a leadership role as Board Member of the Fertility Alliance (charity).

**TRIAL REGISTRATION NUMBER:**

N/A.

WHAT DOES THIS MEAN FOR PATIENTS? This study aimed to calculate the chances of pregnancy in the first year after being diagnosed with fertility problems with any underlying cause.

One in six heterosexual couples are unable to get pregnant within 12 months of stopping contraception and are considered to have fertility issues. Although some are still able to conceive naturally over time, they are usually offered IVF if the initial measures aimed at treating known problems do not work. The chance of conceiving naturally can be very different for different couples. Without tools to estimate the chances of natural conception with different causes of fertility problems and to understand the added value of IVF, there often is a push for early treatment, which can be invasive, expensive, and, sometimes, not yet needed.

Prediction tools that use individual patients’ information are often used to calculate a couple’s chances of conceiving and having a baby. However, the available prediction tools do not take into account all different types of fertility problems. We examined information from 7086 couples diagnosed with all types of fertility problems at a single large UK fertility clinic and found that within a year, about one in eight (13%) of these couples became pregnant and had a baby without treatment. In addition, our new prediction tool is able to provide couples with personalized estimates of the likelihood of conceiving naturally and having a baby, based on their age and specific type of fertility problem. This could help couples and their doctors make more informed decisions about whether, and when, to pursue treatment.

## Introduction

Globally, fertility rates are falling (Bhattacharjee *et al.*, 2024), as are total fertility rates across the UK (National Records of Scotland, 2025; Population Health Monitoring Group, 2025), with one in six couples experiencing difficulty conceiving (Cox *et al.*, 2022). While some couples, particularly those with no clear cause found on routine investigations (‘unexplained’ infertility), may conceive naturally, individual chances vary considerably based on their diagnosis and other characteristics (Eijkemans *et al.*, 2008; McLernon *et al.*, 2016; ElMokhallalati *et al.*, 2019).

Current treatment protocols for all types of infertility typically progress to IVF if initial measures aimed at correcting any known underlying problems are unsuccessful. Where no cause is found, couples are encouraged to try on their own for a period of time before considering active treatment such as IUI or IVF to bypass any unidentified problems. Ideally, in these cases, treatment decisions should be informed by the additional value of these interventions above the couples’ background chance of natural conception. Without clear prognostic information, early intervention with ART can potentially lead to overtreatment at considerable emotional, clinical, and economic costs (Kersten *et al.*, 2015). Evidence-based decision-making is critical to prioritize access to those least likely to conceive naturally.

Several prediction models have been developed to estimate the chance of natural conception in infertile couples, primarily for those with unexplained infertility. For example, the Hunault *et al.* (2004) model, used as part of an initial assessment in the Netherlands, estimates the chance of natural conception in the first year after diagnosis (Hunault *et al.*, 2004). Those with a <30% predicted probability of conception in that time are considered eligible for funded treatment, i.e. IUI with ovarian stimulation (IUI-OS) or IVF (NVOG, 2020).

Later, van Eekelen *et al.* (2017) extended this model to predict chances of conception over 12 months at multiple time points throughout a couple’s treatment journey (up to 18 months from initial assessment) (van Eekelen *et al.*, 2017). In 2019, McLernon *et al.* (2019) published a dynamic prediction model that could estimate prognosis in terms of treatment-dependent and treatment-independent pregnancy leading to live birth in couples with unexplained subfertility.

While all of these models primarily focus on unexplained infertility, couples with underlying causes of infertility (with the exception of those with azoospermia and bilateral absent fallopian tubes) also have a measurable (albeit lower) chance of natural conception (Shimizu *et al.*, 1999; Eijkemans *et al.*, 2008; Khalili *et al.*, 2012; Pandey *et al.*, 2014). Although an awareness of their chances of natural conception leading to live birth would be helpful in informing a decision about seeking assisted reproduction, there are no existing models that can help couples and policymakers gauge the additional value of invasive and potentially expensive fertility treatments.

The North-East of Scotland is a region with a single fertility clinic offering secondary- and tertiary-level care to all infertile couples. The ability to link fertility to maternity data has allowed researchers a unique opportunity to determine natural conception rates in an infertile population (DoPierala *et al.*, 2016). In this study, our objective was to develop a clinical prediction model to estimate the chances of natural conception over a 12-month period in all couples attending a fertility clinic, regardless of their underlying causes of infertility.

## Materials and methods

### Ethical approval

Approval for this study was granted by the North Node Privacy Advisory Committee (Study ID 6-106-21).

### Study population

This population-based retrospective cohort study included all couples who registered at the Aberdeen Fertility Clinic (AFC) between 1998 and 2016, followed up for a minimum of 2 years to detect live birth outcomes. This tertiary clinic covers the entire Grampian population in the North-East of Scotland. All couples underwent routine fertility investigations, including semen analyses, midluteal progesterone, and tubal evaluation. The diagnostic work-up period was assumed to take around 30 days for those with anovulatory infertility and 3 months for all others to allow time for full investigation, including assessment of tubal patency where applicable (as this may require waiting for diagnostic laparoscopy).

Clinical and diagnostic information of all women was linked with available treatment and pregnancy outcome data to determine the chances of natural conception leading to live birth. Couples registering after 2016 were not included as data collection in the AFC database is not currently complete beyond this point.

We excluded couples who did not give consent for their data to be used for research purposes; couples not residing in Grampian who may not have had birth outcomes recorded and therefore been lost to follow-up; and couples where the female partner was aged under 18 or over 50. We also excluded a small number of couples who started treatment before the end of the assumed diagnostic work-up period, as this early intervention precluded any opportunity to observe a natural conception leading to live birth.

### Data sources

The NHS Grampian AFC Database (AFCD) collects data from all subfertile couples who live in Grampian, Scotland, and who undergo fertility investigations or treatment (both NHS and self-funded). This database is audited regularly for validation purposes and contains patient and treatment characteristics for all fertility patients in the region, with data available until 2016. This database was then linked to two local databases containing pregnancy outcomes, based on national identifiers. The NHS Grampian Aberdeen Maternity and Neonatal Databank (AMND) has gathered information on all pregnancy events and births in Aberdeen City from 1951 to 2017. Completeness, validity, and reliability are regularly checked, with studies showing that data from 1986 are 99% accurate (Bhattacharya and Campbell, 2005). The database holds the records of ∼200 000 pregnancies. Badgernet is an electronic maternity and neonatal patient record system used in many areas across the UK. In NHS Grampian, from 2017, perinatal patient data from all local hospitals have been captured on Badgernet by all professionals involved in patient care. This electronic patient record system allows storage, coding, and extraction of perinatal data for research. Data from these three resources were extracted, linked, and pseudo-anonymized by the Grampian Data Safe Haven at the University of Aberdeen. De-identified data were then released to the researchers for analyses within the Data Safe Haven to ensure the security of the data.

### Outcome

We predicted the chance of natural conception within the first year after diagnosis, leading to live birth, in a population of couples diagnosed with any cause of infertility. The key timepoint for making predictions was at completion of diagnostic investigations (∼3 months after first registration at the fertility clinic, as per the method used by McLernon *et al.* (2019), limited to 30 days for those with anovulatory infertility). These live births were identified as natural pregnancies if the estimated start date for the pregnancy (calculated by subtracting the gestational age at delivery from the date of delivery) occurred prior to the date of first fertility treatment (inclusive of ovulation induction [OI], artificial insemination [IUI], and IVF/ICSI).

### Statistical analyses

To assess the characteristics of the baseline population, we generated descriptive statistics of couples at the point of first registration at the clinic. Median time to live birth was calculated from the end of the diagnostic work-up period. Both female BMI and duration of infertility were winsorized (values over the 99th percentile were truncated to the 99th percentile) to minimize the impact of extreme values on modelling.

As our timeframe of interest was the first year after diagnosis, we administratively censored the data at 365 days from the end of the diagnostic work-up period. We tested the proportional hazards assumption (i.e. the assumption that the hazards of the outcome are comparable across different levels of each predictor over the follow-up period) using the Grambsch–Therneau test statistic (Therneau and Grambsch, 2000).

#### Prediction modelling

A Cox proportional hazards model was developed to estimate the chance of natural conception over the first year from diagnosis of infertility, where the pregnancy led to a live birth.

Couples who did not have the event were censored at the first occurrence of any of the following events: starting treatment (inclusive of OI, IUI, and IVF/ICSI); returning to the clinic with a new partner; or the end of the study period.

Covariates used in the prediction model included: year of first registration at the fertility clinic; duration of infertility; age of the female partner; female BMI; female smoking (ever vs never); female alcohol use (any vs none); male factor infertility; endometriosis; ovulatory infertility; unexplained infertility; and tubal infertility. These have been identified in previous studies as key predictors for both treatment-dependent and independent conception (Eijkemans *et al.*, 2008; McLernon *et al.*, 2016; ElMokhallalati *et al.*, 2019).

Continuous variables were assessed for non-linearity with the predicted outcome and, where such was evident, were included in the model as restricted cubic spline functions (see Supplementary File S1).

A Cox proportional hazards model including patient characteristics and type of infertility, as above, was used to estimate the individualized chance of natural conception (within 1 year from diagnosis) leading to live birth. All clinically relevant predictors were entered into the model at once. The hazard ratios and 95% CIs for each predictor were calculated. Predictors included in the model using restricted cubic splines were interpreted using interquartile hazard ratios (comparing the 75th percentile value with the 25th percentile value) to aid interpretation.

#### Model performance

We assessed the performance of the model within the dataset used to develop it (apparent validation) and within the underlying population (internal validation). Performance was assessed using methods for discrimination and calibration. To assess discrimination (i.e. the model’s ability to distinguish between patients with a high chance of live birth following natural pregnancy and those with a low chance), Uno’s c-statistic and time-dependent area under the curve were used. Both methods account for censoring with time-dependent weighting (Uno *et al.*, 2007; Uno *et al.*, 2011). Calibration statistics compare the difference between the model’s predictions and the observed outcomes of the population studied. We used mean calibration (1−Kaplan–Meier survival fraction at 1 year divided by the average predicted chance of conception at 1 year leading to live birth) to assess global under- or over-prediction and calibration slope to assess whether low probabilities of live birth are too low and high probabilities are too high (or vice versa) (McLernon *et al.*, 2023). As an overall assessment of performance, we calculated the Brier score and the Index of Prediction Accuracy (IPA). The Brier score represents the mean squared difference between observed events and the model-predicted probability of natural conception at 1 year from diagnosis. Since the maximum Brier score varies by event rate, it is easier to interpret a scaled Brier score ranging from 0 to 100%. This is calculated as 100×(1 − (Brier score of the developed model divided by Brier score of the null model)).

Internal validation of the model was conducted with bootstrap resampling to assess how well the model may predict within the underlying population. This allows us to adjust the performance metrics for any over-optimism in apparent performance. We used 200 bootstrap samples with replacement, each with the same sample size as the original dataset. The model development process was repeated in each bootstrap sample. For each of these 200 models, performance was assessed in the bootstrap sample (bootstrap performance) and in the original dataset (test performance). The difference between the bootstrap and test performance was averaged across the 200 samples, and this represents the optimism in performance. The optimism-corrected performance was calculated as the apparent performance minus the optimism.

#### Clinical usefulness

To assess clinical utility and impact on decision-making, we performed a decision curve analysis to quantify the net benefit of using the model across a range of risk thresholds (where a risk threshold represents a percentage chance of natural pregnancy leading to live birth, i.e. where the risks of treatment would be justified) (Vickers *et al.*, 2016). Net benefit is the weighted sum of true positives (those classified correctly by the model as being unlikely to achieve a live birth without treatment within the first year after diagnosis) and false positives (those classified incorrectly, i.e. the model predicted they were unlikely to achieve live birth from a natural pregnancy, but in fact they did). These ‘false positives’ are particularly clinically relevant, as they represent couples who, if the model were used to determine eligibility for treatment, may receive unnecessary fertility treatment.

We plotted the net benefit of the model against alternative strategies of ‘treat all’ and ‘treat none’ to determine whether use of the model improved decision-making. We then compared the difference in net benefit between ‘treat all’ and our model, at risk thresholds of <10%, 20%, and 30% chance of natural conception within 1 year after diagnosis leading to live birth. This approach compares the expected proportion of true positives (patients correctly identified as likely to benefit from treatment) minus the proportion of false positives (patients incorrectly identified, who would be treated unnecessarily) weighted by the relative harm of an incorrect classification. More detail on our approach is contained in Supplementary File S2.

All analyses were conducted in line with guidance published by the international STRengthening Analytical Thinking for Observational Studies (STRATOS) initiative (McLernon *et al.*, 2023), as well as TRIPOD + AI guidance (Collins *et al*., 2024).

#### Sensitivity analyses

We generated a further model using inverse probability of censoring weighting (van Geloven *et al.*, 2014) to test whether there was a difference in model parameters or fit when accounting for the competing risk of starting fertility treatments (Supplementary File S3).

All analyses were performed using R (version 4.4.2, R Foundation for Statistical Computing, Vienna, Austria).

## Results

### Baseline characteristics

Between 1998 and 2015, 9757 infertile couples registered for the first time at AFC. After application of exclusion criteria (Supplementary Fig. S1), 7086 of them were included in the study. Their clinical characteristics at the time of first registration are shown in Table 1. The median duration of infertility was 2 years; the mean age of the female partner was 32.3 years (SD 5.4); and 4055 (58%) and 3932 (56%) of male and female partners, respectively, had no previous pregnancies. Male factor infertility was the most common subtype of infertility, present in 1913 couples (31%).

**Table 1.** Baseline characteristics of all couples on first registration at the fertility clinic.

Characteristic	**Couples (n = 7086)** ^1^
**Age of female partner (years), mean (SD)**	32.3 (5.4)
**Duration of infertility (years), median (IQR)**	2.00 (1.33, 3.00)
*Missing*, *n*	*580*
**History of previous pregnancy—female partner**	3098 (44%)
*Missing*, *n*	*56*
**History of previous pregnancy—male partner**	2898 (42%)
*Missing*, *n*	*133*
**Smoking history in female partner (ever)**	1550 (23%)
*Missing*, *n*	*198*
**Alcohol use in female partner (any)**	5214 (75%)
*Missing*, *n*	*155*
**Female BMI (kg/m^2^), median (IQR)**	24.6 (22.0, 29.0)
*Missing*, *n*	*1278*
**Male factor infertility**	1913 (31%)
Of these, natural conception leading to live birth, n	176
Live birth rate (95% CI)^2^	11.2% (9.6, 12.8)
*Missing*, *n*	*823*
**Endometriosis**	290 (4.6%)
Of these, natural conception leading to live birth, n	26
Live birth rate (95% CI)^2^	12% (7.5, 16.3)
*Missing*, *n*	*810*
**Anovulation**	1614 (26%)
Of these, natural conception leading to live birth, n	199
Live birth rate (95% CI)^2^	17.9% (15.6, 20.2)
*Missing*, *n*	*812*
**Unexplained infertility**	1628 (26%)
Of these, natural conception leading to live birth, n	305
Live birth rate (95% CI)^2^	21% (18.8, 23.1)
*Missing*, *n*	*810*
**Tubal infertility**	1151 (18%)
Of these, natural conception leading to live birth, n	92
Live birth rate (95% CI)^2^	9.5% (7.6, 11.3)
*Missing*, *n*	*809*
**Other infertility** ^3^	447 (7.1%)
Of these, natural conception leading to live birth, n	37
Live birth rate (95% CI)^2^	9.2% (6.3, 12)
*Missing*, *n*	*811*

1 n (%) unless otherwise stated.

2 Kaplan–Meier non-parametric estimate of the survival function.

3 Inclusive of cervical factor, uterine malformation, or sexual dysfunction.

IQR, interquartile range.

Overall, 891 (13%) of the population experienced a live birth following a natural conception (i.e. conception within the first year after diagnosis, prior to any fertility treatment) with a median time to conception of 140 days (interquartile range 70–230).

### Missing data

Of all the couples, 34.4% had missing data in any of the key predictors used in the model, as illustrated in Supplementary Table S1. The highest proportions of missing data were observed in female BMI (18.0% missing). Missing data were more common in those who registered in the earliest years of the study and in those with female secondary infertility. Couples with male factor, tubal, or other types of infertility had a higher proportion of missing data than those without; the opposite was observed in couples with anovulatory or unexplained infertility, where missing data were less common compared to couples without these types of infertility.

These data were assumed to be missing at random, and multiple imputation by chained equations was used to impute missing values; 40 imputed datasets were generated (see Supplementary File S4).

### Model development

Year of first registration, female age, BMI, and duration of infertility were all found to have potential non-linear relationships with the outcome and were therefore included as restricted cubic spline functions in the model (Supplementary Fig. S2). Results of the proportional hazard testing are shown in Supplementary Table S2.

### Prediction of natural conception leading to live birth

Table 2 shows the relationship between each predictor and the rate of live birth following natural pregnancy over the 1-year follow-up period, compared against the given reference values. Increasing female age and duration of infertility were negatively associated with the chance of live birth. Similarly, couples where the female partner had a BMI of 29 were 12% less likely over time to conceive naturally in the first year after diagnosis than couples where the female partner had a BMI of 22 (HR 0.88; 95% CI [0.78, 0.98]). While a history of smoking in the female partner was associated with a reduction in the chance of live birth (0.74 [0.62, 0.89]) compared with never-smokers, a history of any alcohol use was not a significant predictor (1.08 [0.91, 1.28]).

**Table 2.** Effect and importance (in descending order) of each couple characteristic and diagnosis on the chance of live birth resulting from natural conception in the first year from diagnosis.

Characteristic (female partner unless specified)	**HR (95% CI)** ^1^	*P*-value
Age of female partner (years) (36 vs 28)^2^	0.62 (0.55, 0.70)	<0.0001*
Duration of infertility, winsorized (years) (3 vs 1.3)^2^	0.64 (0.57, 0.72)	<0.0001*
Female history of previous pregnancy (yes vs no)	1.30 (1.12, 1.50)	<0.001*
Smoking history (ever) (yes vs. no)	0.74 (0.62, 0.89)	0.001*
Female BMI—winsorized (kg/m^2^) (28.8 vs 21.9)^2^	0.88 (0.78, 0.98)	0.02*
Year of first registration (2012 vs 2003)^2^	1.09 (0.96, 1.23)	0.12
History of alcohol use (ever) (yes vs no)	1.08 (0.91, 1.28)	0.40
**Diagnosis of infertility** ^3^		
Tubal infertility (yes vs no)	0.62 (0.47, 0.81)	<0.001*
Male factor infertility (yes vs no)	0.71 (0.57, 0.90)	<0.01*
Other infertility^4^ (yes vs no)	0.68 (0.50, 0.92)	0.014*
Unexplained infertility (yes vs no)	1.29 (1.00, 1.67)	0.05
Anovulatory infertility (yes vs no)	1.12 (0.87, 1.43)	0.40
Endometriosis (yes vs no)	0.83 (0.55, 1.24)	0.40

1 HR, hazard ratio (adjusted) pooled over 40 imputations.

2 Variables fitted as restricted cubic splines are presented as interquartile hazard ratios to ease interpretation—i.e. the hazard of live birth after natural conception for the 75th percentile vs the 25th percentile value.

3 Reference group for all diagnoses is all couples without that diagnosis.

4 Inclusive of cervical factor, uterine malformation, or sexual dysfunction.

* Statistically significant difference at *P* < 0.05, tested using Wald test.

A diagnosis of unexplained infertility had a positive relationship with the chance of natural pregnancy leading to live birth, increasing the chance by 29% (1.29 [1.00, 1.67]) compared against all couples without unexplained infertility. Those with tubal infertility were 38% less likely to experience a live birth than those without (0.62 [0.47, 0.81]). A diagnosis of male factor infertility also had a negative association with natural conception leading to live birth (0.71 [0.57, 0.90]), but in our population, both endometriosis and anovulatory infertility did not significantly influence the chance of natural conception resulting in live birth when compared against all other couples without these diagnoses.

### Calculating the predicted chance of natural conception leading to live birth by subtype of infertility

Table 3 illustrates the estimates provided by the model for simulated patients across all major subtypes of infertility and a range of ages for the female partner. These estimates are calculated using the baseline risk and coefficients from the prediction model, as shown in Supplementary File S5.

**Table 3.** Examples of the predicted chance of live birth from natural conception within 1 year from diagnosis, by female age and type of infertility in simulated patients.

Type of infertility and female age (years)	Predicted chance of conception leading to live birth (95% CI)
Unexplained	
25	20.7 (16.1, 25.0)
30	20.3 (16.2, 24.2)
35	15.0 (11.8, 18.1)
40	8.6 (6.2, 10.9)
Ovulatory	
25	14.0 (10.6, 17.2)
30	13.7 (10.4, 16.9)
35	10.0 (7.5, 12.5)
40	5.7 (3.9, 7.4)
Tubal	
25	10.4 (7.3, 13.5)
30	10.2 (7.3, 13.1)
35	7.4 (5.3, 9.6)
40	4.2 (2.7, 5.6)
Endometriosis	
25	12.8 (7.5, 17.9)
30	12.6 (7.4, 17.4)
35	9.2 (5.4, 12.8)
40	5.2 (2.8, 7.5)
Male factor	
25	11.8 (8.9, 14.5)
30	11.5 (8.8, 14.1)
35	8.4 (6.4, 10.4)
40	4.7 (3.3, 6.1)
Other	
25	11.5 (7.1, 15.7)
30	11.3 (7.1, 15.3)
35	8.2 (5.1, 11.2)
40	4.6 (2.7, 6.5)
25	11.5 (7.1, 15.7)

For all examples: continuous variables set to mean of baseline population (median used for duration of infertility and BMI), and categorical variables set to 0. ‘Other’ infertility is inclusive of cervical factor, uterine malformation, or sexual dysfunction.

In a couple with unexplained infertility where the age of the female partner is 25, the predicted probability of natural conception at 1-year post-diagnosis (leading to live birth) was 20.7%. This is in contrast with the predicted probability for a couple where the female partner is the same age but has a diagnosis of tubal infertility: the predicted probability is almost half of that of the first couple, at 10.4%.

Further examples of simulated patients with combinations of different characteristics are shown in Supplementary Table S3 to demonstrate the influence of both previous pregnancy and duration of infertility on the predictions. For example, take a couple with primary ovulatory infertility for 3 years where the female partner is aged 25. Their predicted probability is 11.3%. However, a similar couple with ovulatory infertility, where the female partner is also aged 25 but has secondary infertility for only 1 year, has a predicted probability of 23.5%.

A web-based calculator based on this model is freely available at [njcameron.shinyapps.io/LB_calc/], with the full R code hosted publicly at [github.com/nataliejcameron/LB_calc].

### Assessment of model performance

**Figure 1.**
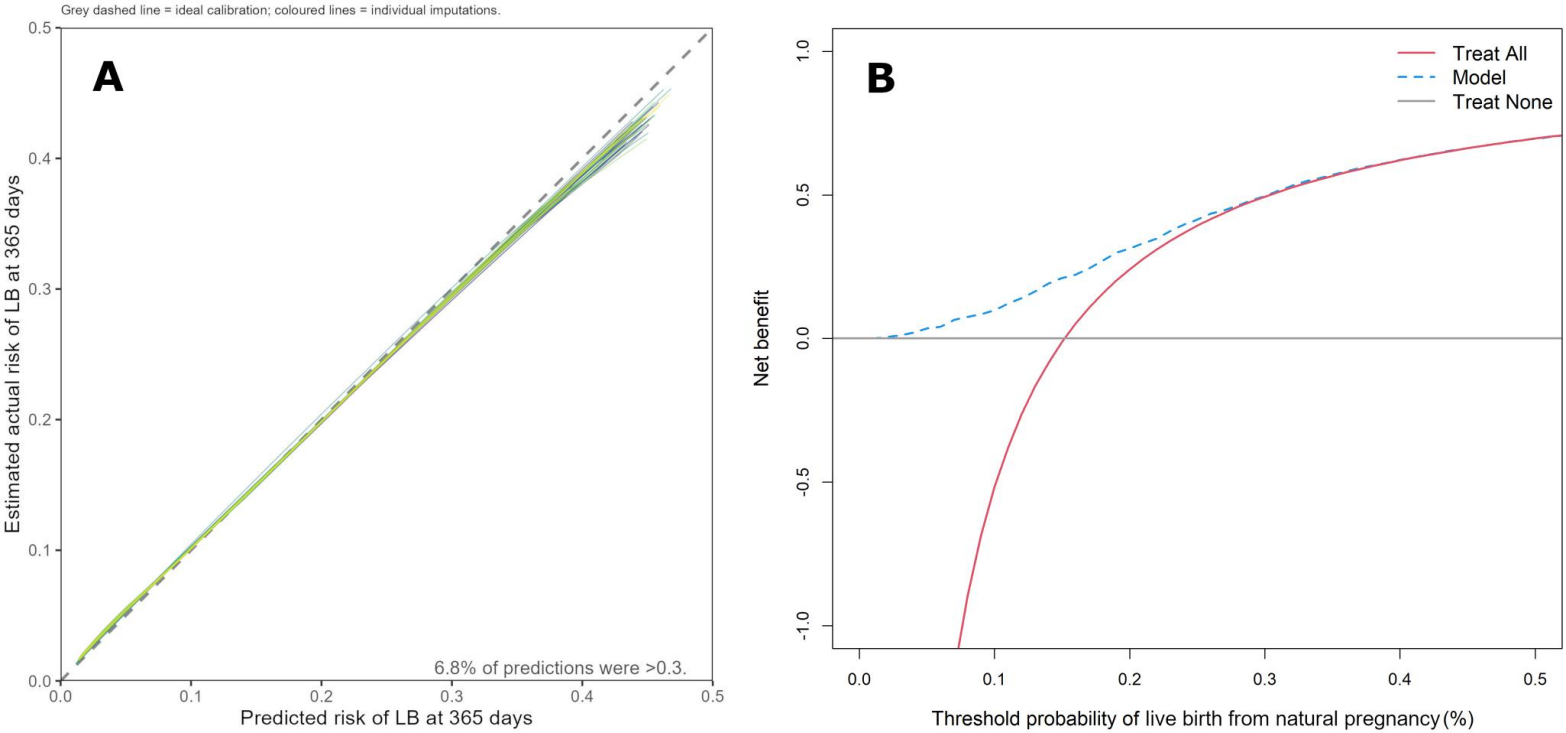
**Reliability and clinical utility of the developed prediction model for natural conception leading to live birth in couples with infertility**. (**A**) Optimism-corrected calibration plot showing correlation between estimated observed chance of natural conception leading to live birth and the predicted probabilities of natural conception leading to live birth from the model, in each imputed dataset. The grey dotted line represents ideal calibration, where predicted risk equals actual risk; the coloured lines represent calibration across each individual imputed dataset. (**B**) Decision curve analysis showing the net benefit of using the model (blue dashed line) to identify couples with a low chance of natural conception leading to live birth across a range of threshold probabilities, compared against treating all (red line) or no patients (grey line). LB, live birth following natural conception.

Table 4 shows the results of the performance analyses, as pooled across the 40 imputed datasets. The mean calibration in the internal validation using bootstrapped samples showed good agreement between the observed outcomes and the average predicted probability, with an O/E of 1.000 (95% CI: 0.991, 1.009). The calibration slope for the model was <1 at 0.949 (0.933, 0.965), indicating minor evidence of overprediction. The calibration plot in Fig. 1A shows that, across all 40 imputations, this overprediction tended to occur within those with a higher predicted probability of live birth >0.3, which corresponded to 6.8% of the predicted probabilities. Supplementary Fig. S3 shows the calibration curve for each of the imputed datasets separately.

**Table 4.** Performance measures assessing the accuracy and reliability of the predictions generated by the model in the dataset used for development (apparent validation) and in the underlying population (internal validation).

**Performance measure**	Apparent validation	**Internal validation: optimism-corrected performance** ^1^
**Calibration**		
Mean calibration (O/E)—fixed	1	1.000 (0.991, 1.009)
Weak calibration (slope)—fixed	1	0.949 (0.933, 0.965)
**Discrimination**		
Uno C-statistic	0.660 (0.643, 0.677)	0.647 (0.644, 0.649)
AUC (at 365 days)	0.672 (0.643, 0.682)	0.655 (0.652, 0.657)
**Overall**		
Brier score (overall)	0.116 (0.111, 0.122)	0.117 (0.116, 0.117)
Scaled Brier score	3.8% (2.6, 5.1)	3.6% (3.4, 3.8)
**Clinical usefulness** ^2^		
Difference in model net benefit and treat-all net benefit at 10% threshold	0.616	NA
Difference in model net benefit and treat-all net benefit at 20% threshold	0.072	NA
Difference in model net benefit and treat-all net benefit at 30% threshold	0.002	NA

Where applicable, figures are presented as estimate (95% CI).

1 The optimism-corrected performance measures from the model developed using the 40 imputed datasets were pooled using a random-effects inverse-variance model (Supplementary File S2)

2 Average across 40 imputed datasets.

O/E, observed/expected; AUC, area under the curve; NA, not applicable.

The apparent c-statistic for the model was 0.660, which is the probability that a randomly selected couple with a given time to live birth has a lower chance of live birth than a randomly selected couple with a shorter time to live birth. When corrected for optimism using internal validation, this estimate was very slightly lower at 0.655 (0.652, 0.657).

The Brier score was 0.116, indicating the predictions generated by the model were close to the observed outcomes with very little change in the optimism-corrected score (0.117). The scaled Brier score showed our model gave a 3.6% improvement in predictive accuracy over a null model once corrected for optimism in internal validation.

Finally, we performed a decision curve analysis pooled across all 40 imputations (Fig. 1B), which showed an improvement in net benefit over the ‘treat-all’ and ‘treat none’ approaches in the range of clinically important risk thresholds (Table 3). At a risk threshold of 20%, the model showed a difference in net benefit of 0.072 over the ‘treat-all approach’. This suggests that, compared against providing fertility treatment to all couples, if only those with a <20% predicted chance of achieving live birth from natural pregnancy within 1 year were treated, ∼7.2 more couples per 100 would be correctly identified as being likely to benefit from fertility treatment after penalizing for the unnecessary treatments in those likely to achieve natural conception.

#### Subgroup analysis of model performance

*Post-hoc* subgroup analysis was performed to assess model performance across different diagnostic categories (Supplementary Table S4). After internal validation, the model demonstrated consistent discriminative ability across the subgroups, with the lowest discriminative ability in couples with endometriosis or other infertility (inclusive of uterine malformation, cervical factor, or sexual dysfunction), with an optimism-corrected Uno’s C-statistic of 0.593 in both subgroups. Tubal infertility had the highest optimism-corrected C-statistic at 0.648 (95% CI: 0.640, 0.656).

Mean calibration (O/E) showed good agreement between observed and predicted outcomes across subgroups, ranging from 0.983 (0.970, 0.996) for unexplained infertility to 1.122 (1.060, 1.188) for those with endometriosis. The calibration slope showed overfitting in the endometriosis and other infertility subgroups, with optimism-corrected slope of 0.592 and 0.601, respectively (Supplementary Table S4). This is corrected for in the model formula (Supplementary File S5) and online calculator by multiplying the prognostic index by the calibration slope when calculating predictions for these subgroups.

## Discussion

This novel prediction model uniquely provided individualized predicted chances, within 1 year of first diagnosis of natural conception leading to live birth, for couples with all common infertility types.

### Strengths and limitations

This is the first article, to the authors’ knowledge, to report a prediction model for rates of natural conception leading to live birth across a population comprising all types of infertility. This is due to our unique population-level cohort from a single NHS clinic that serves an entire region with low levels of migration out of the area, minimizing the risk of loss to follow-up (Ayorinde *et al.*, 2016). There is limited access to longitudinal datasets, containing clinical information of couples assessed for infertility, linking their outcomes from both ART and pregnancies occurring without treatment. There are thus very few opportunities anywhere in the world to predict the individualized chance of natural conception leading to live birth in a population of infertile couples. The NHS setting of this clinic meant that waiting lists and established funding criteria for treatment (i.e. two years’ duration of unexplained infertility) create a natural observation window to observe the baseline rate of natural conception in a population of couples with infertility (National Institute for Health and Care Excellence, 2017).

We have reformatted the model into an online calculator that is freely accessible by patients, the public, and other researchers (https://njcameron.shinyapps.io/LB_calc/). To ensure transparency and reproducibility, we have published open-source code allowing clear understanding of how the calculations are performed (https://github.com/nataliejcameron/LB_calc/).

Our study was limited by data being gathered from a single clinic up until 2016, though temporal trends were addressed by adjusting for year of first registration. The absence of a significant association with natural conception suggests that this relationship has remained stable with time. As more data become available in the future, it would be useful to update or externally validate the model in a more contemporaneous population. Missing data were a limitation in our study, as is often the case with longitudinal retrospective data. We used multiple imputation to address this whilst preserving the sample size and precision.

It is difficult to define an exact date of diagnosis in subfertile couples, as this will vary depending on the clinical history and the timing (and range) of tests performed for each couple. As such, we set the length of the diagnostic workup period to 12 weeks (as previously described by McLernon *et al.* (2019)) or 30 days for patients with ovulatory infertility (as these patients are likely to be diagnosed and commence treatment more quickly). As a result, a small number of early conceptions within this ‘workup’ period are potentially missed from follow-up.

While the strongest known independent predictors of natural conception were included (Hunault *et al.*, 2004; Eijkemans *et al.*, 2008; McLernon *et al.*, 2019; Shingshetty *et al.*, 2022), uncollected data on ovarian reserve, severity of endometriosis, or subtype of ovulatory disorders could not be assessed. As scoring tools such as the Endometriosis Fertility Index become more commonly used, future studies could include this to further refine predictions. We did not include more detailed predictors for male factor infertility in our model, such as male age or semen parameters. Semen analyses are highly variable, even from sample-to-sample within individuals (Leushuis *et al.*, 2010; Leushuis *et al.*, 2014). The relationship between semen parameters and natural conception is similarly inconsistent in the literature (Andersen *et al.*, 2002; Eijkemans *et al.*, 2008; Buck Louis *et al.*, 2014; Lamb and Marinaro, 2023).

### Comparison with the literature

Our observed rate of natural conception leading to live birth within 1 year from diagnosis was 13%, in alignment with data from Collins *et al.* (1995) showing a live birth rate of 14.3% in a Canadian population of couples with untreated infertility of varying types and severity. This contrasts with the estimate of the ongoing pregnancy rate from Eijkemans *et al.* (2008) at 9.1% at 1 year, based on a population of patients on an IVF waiting list who have already met the referral criteria for IVF and are likely to have a poorer prognosis. Conversely, the Hunault model, based on mild male or unexplained infertility (after excluding patients with tubal infertility, anovulation and azoospermia), estimated natural conception rates of 18–37% (Hunault *et al.*, 2004). The discriminative ability of our model is analogous to previously published models (Hunault *et al.*, 2004; Eijkemans *et al.*, 2008; van Eekelen *et al.*, 2017; McLernon *et al.*, 2019).

### Interpretation

We have shown that a range of couple characteristics influence the chance of natural conception leading to live birth, including but not limited to the specific diagnosis. The model’s hazard ratios illustrate the influence of each characteristic on the rate of this outcome occurring over the entire follow-up period. Diagnoses such as endometriosis and ovulatory infertility did not have a statistically significant influence on this rate, potentially due to the large range of clinical severities of these conditions. Those with a high burden of clinical disease in endometriosis are known to have a poorer prognosis for natural conception than those with minimal disease (Adamson and Pasta, 2010). Regardless of statistical significance, information on these diagnoses will contribute to overall model fit and predictive performance. The model is still useful for patients with ovulatory infertility and endometriosis, but their other characteristics such as age and duration of infertility may have greater influence than their diagnosis. Compared to models limited to unexplained infertility, our model provides a more flexible estimate, given that in clinical practise a couple’s prognosis may be a sum of multiple overlapping infertility diagnoses as well as their baseline characteristics. The internally validated C-statistic of our model is very similar to models published previously in unexplained infertility (Hunault *et al.*, 2004; van Eekelen *et al.*, 2017; McLernon *et al.*, 2019). This suggests that including the broader population of all couples with infertility does not compromise its ability to distinguish between those with a high and low chance of natural conception leading to live birth. Subgroup analysis of model performance by type of infertility showed a range of optimism-corrected C-statistics from 0.593 (endometriosis, other infertility) to 0.648 (tubal infertility), again comparable to models published in unexplained infertility only.

The model showed very good calibration in the underlying population. There was some evidence of overprediction in couples with higher chances of conception (over 30%), which reflects only 6.8% of the study population. For the range of clinically relevant risk thresholds where decisions could potentially be made regarding treatment, i.e. 10–30%, the model shows excellent agreement between predicted and observed live birth. In subgroups of couples with endometriosis or other infertility, there was some evidence of overfitting in the calibration slope, which is corrected for by applying the slope as a shrinkage factor when calculating predictions for couples with these diagnoses. The model showed fair discrimination with a C-statistic of 0.66, which is common in studies predicting live birth. This may reflect the relative homogeneity of the study population, i.e. infertile women of reproductive age, or the absence of key prognostic factors, either unavailable or yet to be identified (Coppus *et al.*, 2009). However, a low C-statistic alone does not imply limited clinical utility. Calibration is arguably more important as models inform couples of their evidence-based chances of success. The decision curve analysis also showed that the model has clinical utility at the above risk thresholds.

### Clinical relevance

There is a global tendency to classify all infertile couples as sterile and to rapidly intervene with expensive and invasive assisted reproduction technology. Our study challenges this mindset by demonstrating that couples with diverse types of infertility often retain a potential for natural conception, shaped by multiple aspects of their baseline characteristics.

The prognostic estimates from this model offer a context-based individual approach towards assessing the net benefit of fertility treatment over and above baseline potential to conceive. IVF, with a 31% live birth rate per embryo transferred in the UK (HFEA, 2024), has been the dominant pathway for fertility treatment but NICE guidance has stressed the importance of assessing its added value (National Institute for Health and Care Excellence, 2017, 2025) for both patients and policymakers. Our model, while developed in a different population, could provide a useful estimate of baseline potential for natural conception to contextualize the chance of treatment-related live birth from existing IVF prediction models (McLernon *et al.*, 2016).

Our model can refine decision-making by accounting for important clinical characteristics and diagnosis to estimate the chance of natural conception. The online calculator (https://njcameron.shinyapps.io/LB_calc/) is freely accessible with open access code for transparency and can be used as an informational aid to augment patient–clinician discussions about prognosis. For example, tubal infertility is consistently associated with a low predicted chance of live birth resulting from natural conception across all ages in our model, such that intervention with ART would almost always be justified. Alternatively, consider a couple with unexplained infertility who have been trying for 1 year, where the female partner is aged 25 and has a history of a previous pregnancy. They would have a predicted 33.8% chance of natural conception within the first year of diagnosis leading to live birth. With counselling, this might support a period of expectant management. For a similar couple with the female partner aged 35 and a duration of infertility of 3 years, the predicted chance falls to 15.3%. By this point, the diminishing chance of live birth may favour intervention with ART.

Because some couples strongly desire natural conception, expectant management carries the risk of psychological distress if pregnancy does not occur (Porter and Bhattacharya, 2008). Patients who have made the decision to end treatment after unsuccessful IVF have emphasized a lack of individualized prognostic information throughout their journey as a key factor in their difficulty in coming to terms with unresolved childlessness (Peddie *et al.*, 2005). Expectations can be balanced with personalized estimates from our model, and where the prognosis is poor, it can help to direct patients towards earlier intervention to avoid delays in necessary care and support realistic decision-making.

At an organizational level, financial constraints must be a consideration in healthcare policy. A clearer identification of subgroups with little chance of natural conception may help policymakers to improve access for these couples who will have the greatest net benefit from fertility treatment.

### Further research

External validation is essential to improve the generalizability of this tool, both in separate populations of infertile populations and more recent cohorts. At the time of writing, the authors are not aware of any comparable external datasets containing information on untreated, natural pregnancies in couples with all types of infertility. The full model formula and calculator are published in an open-source format (https://github.com/nataliejcameron/LB_calc/), explicitly to enable and encourage external validation in other centres should suitable comparable datasets become available.

Beyond this, the value of prediction models as decision-making tools would need to be subjected to robust evaluation in terms of their clinical and cost effectiveness before widespread implementation is considered.

## Conclusion

This is the first prediction model to provide individualized estimates of the chance of natural conception (leading to live birth) during the first year from diagnosis for a population of patients with all types of infertility. This information can help couples and clinicians to augment their understanding of their prognosis at the time of diagnosis of infertility, before making key treatment decisions.

## Supplementary Material

hoag056_Supplementary_Data

## Data Availability

The data underlying this article cannot be shared publicly for the privacy of the individuals who participated in the study. For study protocol or analytical code, please contact the corresponding author.
