# Supplementary material for: Predicting natural conception leading to live birth for couples with infertility: a single-centre population-based cohort study of 7086 couples
Source: Hum Reprod Open. 2026 Jun 13;2026(3):hoag056. doi: 10.1093/hropen/hoag056 (PMC13353215; doi:10.1093/hropen/hoag056)
Supplement: hoag056_Supplementary_Data [file hoag056_supplementary_data.zip › Supplementary_Table_S1.docx]

**Supplementary Table S1:** Baseline characteristics at first clinic registration, by whether couples had any missing data in any of key predictors to be used in prediction models^†^.

| **Characteristic** | **Missing data *n* = 2,443*^1^*** | **No missing data *n* = 4,643*^1^*** | ***p*-value*^2^*** |
| --- | --- | --- | --- |
| Year of first registration |  |  | <0.001* |
| 1998 | 205 (8.4%) | 193 (4.2%) |  |
| 1999 | 205 (8.4%) | 158 (3.4%) |  |
| 2000 | 169 (6.9%) | 148 (3.2%) |  |
| 2001 | 152 (6.2%) | 154 (3.3%) |  |
| 2002 | 111 (4.5%) | 212 (4.6%) |  |
| 2003 | 136 (5.6%) | 205 (4.4%) |  |
| 2004 | 138 (5.6%) | 199 (4.3%) |  |
| 2005 | 128 (5.2%) | 252 (5.4%) |  |
| 2006 | 83 (3.4%) | 242 (5.2%) |  |
| 2007 | 98 (4.0%) | 282 (6.1%) |  |
| 2008 | 75 (3.1%) | 314 (6.8%) |  |
| 2009 | 84 (3.4%) | 397 (8.6%) |  |
| 2010 | 69 (2.8%) | 362 (7.8%) |  |
| 2011 | 69 (2.8%) | 237 (5.1%) |  |
| 2012 | 124 (5.1%) | 367 (7.9%) |  |
| 2013 | 182 (7.4%) | 358 (7.7%) |  |
| 2014 | 174 (7.1%) | 351 (7.6%) |  |
| 2015 | 241 (9.9%) | 212 (4.6%) |  |
| Age of female partner (years), mean (SD) | 32.7 (5.7) | 32.0 (5.2) | <0.001* |
| Age of male partner (years), mean (SD) | 35 (7) | 35 (6) | 0.009* |
| *Missing* | *106* | *0* |  |
| Duration of infertility (years), median (IQR) | 2.00 (1.33, 3.00) | 2.00 (1.33, 3.00) | 0.005* |
| *Missing* | *580* | *0* |  |
| Female secondary infertility | 1,228 (51%) | 1,870 (40%) | <0.001* |
| *Missing* | *56* | *0* |  |
| Smoking history in female partner (ever) | 620 (28%) | 930 (20%) | <0.001* |
| *Missing* | *198* | *0* |  |
| Alcohol use in female partner (any) | 1,753 (77%) | 3,461 (75%) | 0.06 |
| *Missing* | *155* | *0* |  |
| Female BMI (kg/m2), median (IQR) | 24.8 (21.9, 28.7) | 24.6 (22.0, 29.0) | 0.8 |
| *Missing* | *1,278* | *0* |  |
| Male factor infertility | 543 (34%) | 1,370 (30%) | 0.003* |
| *Missing* | *823* | *0* |  |
| Endometriosis | 69 (4.2%) | 221 (4.8%) | 0.4 |
| *Missing* | *810* | *0* |  |
| Anovulation | 254 (16%) | 1,360 (29%) | <0.001* |
| *Missing* | *812* | *0* |  |
| Unexplained infertility | 289 (18%) | 1,339 (29%) | <0.001* |
| *Missing* | *810* | *0* |  |
| Tubal infertility | 431 (26%) | 720 (16%) | <0.001* |
| *Missing* | *809* | *0* |  |
| Other infertility^3^ | 181 (11%) | 266 (5.7%) | <0.001* |
| *Missing* | *811* | *0* |  |
| *^1^*n (%) unless otherwise stated. | | | |
| ^2^Pearson's chi-squared test; Wilcoxon rank sum test | | | |
| ^3^Inclusive of cervical factor, uterine malformation or sexual dysfunction. | | | |
| ^*^ Statistically significant difference at *p*<0.05 | | | |
| ^†^Missing data was more common in those who registered in the earliest years of the study, and in those with secondary infertility. There were significant differences in the types of infertility between those with any missing data and those without: couples with male factor; tubal or other types of infertility were more likely to have some missing data than those without. *Abbreviations:* SD = standard deviation; IQR = interquartile range; BMI = body mass index. | | | |
