# Supplementary material for: Predicting natural conception leading to live birth for couples with infertility: a single-centre population-based cohort study of 7086 couples
Source: Hum Reprod Open. 2026 Jun 13;2026(3):hoag056. doi: 10.1093/hropen/hoag056 (PMC13353215; doi:10.1093/hropen/hoag056)
Supplement: hoag056_Supplementary_Data [file hoag056_supplementary_data.zip › Supplementary_Table_S3.docx]

**Supplementary Table S3:** Examples of calculated predicted chance of natural conception leading to live birth in simulated couples, with different combinations of key prognostic factors.

| **Previous pregnancy history (female)** | **Age of female partner (years)** | **Duration of infertility (years)** | **Predicted chance of live birth, % (95% confidence interval)** |
| --- | --- | --- | --- |
| ***Unexplained infertility*** | | | |
| **No** | 25 | 1 | 27.6 (21.5, 33.2) |
|  |  | 3 | 16.9 (12.9, 20.7) |
|  | 30 | 1 | 27.1 (21.5, 32.3) |
|  |  | 3 | 16.6 (13.0, 20.0) |
|  | 35 | 1 | 20.3 (15.9, 24.4) |
|  |  | 3 | 12.2 (9.5, 14.8) |
| **Yes** | 25 | 1 | 33.8 (26.3, 40.5) |
|  |  | 3 | 21.0 (16.0, 25.8) |
|  | 30 | 1 | 33.2 (26.5, 39.3) |
|  |  | 3 | 20.6 (16.2, 24.9) |
|  | 35 | 1 | 25.1 (19.9, 29.9) |
|  |  | 3 | 15.3 (12.0, 18.4) |
| ***Ovulatory infertility*** | | | |
| **No** | 25 | 1 | 18.9 (14.6, 23.1) |
|  |  | 3 | 11.3 (8.4, 14.2) |
|  | 30 | 1 | 18.6 (14.3, 22.6) |
|  |  | 3 | 11.1 (8.3, 13.9) |
|  | 35 | 1 | 13.7 (10.3, 16.9) |
|  |  | 3 | 8.1 (5.9, 10.2) |
| **Yes** | 25 | 1 | 23.5 (18.0, 28.6) |
|  |  | 3 | 14.2 (10.4, 17.9) |
|  | 30 | 1 | 23.1 (17.8, 28.0) |
|  |  | 3 | 14.0 (10.3, 17.4) |
|  | 35 | 1 | 17.1 (13.0, 21.0) |
|  |  | 3 | 10.2 (7.5, 12.9) |
| ***Tubal infertility*** | | | |
| **No** | 25 | 1 | 14.2 (9.9, 18.4) |
|  |  | 3 | 8.4 (5.8, 11.0) |
|  | 30 | 1 | 14.0 (9.9, 17.8) |
|  |  | 3 | 8.3 (5.8, 10.6) |
|  | 35 | 1 | 10.2 (7.2, 13.1) |
|  |  | 3 | 6.0 (4.2, 7.7) |
| **Yes** | 25 | 1 | 17.8 (12.6, 22.7) |
|  |  | 3 | 10.6 (7.4, 13.7) |
|  | 30 | 1 | 17.4 (12.7, 21.9) |
|  |  | 3 | 10.4 (7.5, 13.2) |
|  | 35 | 1 | 12.8 (9.3, 16.2) |
|  |  | 3 | 7.6 (5.5, 9.6) |
| ***Endometriosis*** | | | |
| **No** | 25 | 1 | 17.4 (10.2, 24.0) |
|  |  | 3 | 10.4 (5.9, 14.6) |
|  | 30 | 1 | 17.1 (10.2, 23.5) |
|  |  | 3 | 10.2 (5.9, 14.2) |
|  | 35 | 1 | 12.6 (7.4, 17.4) |
|  |  | 3 | 7.4 (4.3, 10.4) |
| **Yes** | 25 | 1 | 21.7 (12.6, 29.8) |
|  |  | 3 | 13.1 (7.4, 18.4) |
|  | 30 | 1 | 21.2 (12.6, 29.0) |
|  |  | 3 | 12.8 (7.4, 17.9) |
|  | 35 | 1 | 15.7 (9.3, 21.7) |
|  |  | 3 | 9.3 (5.4, 13.1) |
| ***Male factor infertility*** | | | |
| **No** | 25 | 1 | 16.0 (12.0, 19.8) |
|  |  | 3 | 9.5 (7.1, 11.9) |
|  | 30 | 1 | 15.7 (11.9, 19.3) |
|  |  | 3 | 9.3 (7.1, 11.5) |
|  | 35 | 1 | 11.5 (8.6, 14.3) |
|  |  | 3 | 6.8 (5.1, 8.5) |
| **Yes** | 25 | 1 | 20.0 (14.9, 24.7) |
|  |  | 3 | 12.0 (8.8, 15.0) |
|  | 30 | 1 | 19.6 (14.9, 24.0) |
|  |  | 3 | 11.7 (8.8, 14.5) |
|  | 35 | 1 | 14.4 (10.9, 17.8) |
|  |  | 3 | 8.6 (6.4, 10.6) |
| ***Other infertility*** | | | |
| **No** | 25 | 1 | 15.7 (9.7, 21.2) |
|  |  | 3 | 9.3 (5.7, 12.8) |
|  | 30 | 1 | 15.4 (9.6, 20.7) |
|  |  | 3 | 9.1 (5.6, 12.5) |
|  | 35 | 1 | 11.3 (7.0, 15.3) |
|  |  | 3 | 6.6 (4.1, 9.1) |
| **Yes** | 25 | 1 | 19.5 (12.1, 26.3) |
|  |  | 3 | 11.7 (7.1, 16.1) |
|  | 30 | 1 | 19.1 (12.1, 25.6) |
|  |  | 3 | 11.5 (7.1, 15.7) |
|  | 35 | 1 | 14.1 (8.9, 19.0) |
|  |  | 3 | 8.4 (5.2, 11.4) |
| For all examples: continuous variables set to mean of baseline population (median used for BMI), and categorical variables set to 0. 'Other' infertility is inclusive of cervical factor, uterine malformation or sexual dysfunction. | | | |
