# Supplementary material for: Predicting natural conception leading to live birth for couples with infertility: a single-centre population-based cohort study of 7086 couples
Source: Hum Reprod Open. 2026 Jun 13;2026(3):hoag056. doi: 10.1093/hropen/hoag056 (PMC13353215; doi:10.1093/hropen/hoag056)
Supplement: hoag056_Supplementary_Data [file hoag056_supplementary_data.zip › NewSuppFiles.docx]

Supplementary File S1

Model development – restricted cubic splines

To assess for a potential non-linear relationship with the outcome of interest (live birth following natural conception), we fitted each continuous predictor in a univariate Cox regression model. We then plotted the observed and predicted values for the chance of live birth following natural conception against the range of predictor values, and where a non-linear relationship was observed we then refitted the univariate models with a restricted cubic spline function (Supplementary Figure S2) and repeated this over multiple variations of numbers of knots. The number of knots for each variable was chosen based on which provided the lowest value for Akaike’s Information Criterion (AIC).

Duration of infertility was found to have the lowest AIC value at 4 knots, however on fitting the full model this variable was found to have an extremely high coefficient, which was addressed by refitting with three knots.

The models were then refitted and pooled across all 40 imputed datasets to estimate the most appropriate position for the knots to be fitted at, and these were then saved in the dataset.

Supplementary File S2

Assessment of the model’s predictive ability

Methods for pooling performance measures

The optimism-adjusted performance measures from the models developed using the 40 imputed datasets were pooled using a random-effects inverse-variance model.

Since ratios like O/E are naturally skewed they have a sampling distribution that is closer to normal on the log scale. We took the natural log transformation of the O/E values before running the meta-analysis. The standard errors were also transformed using the delta method. The pooled estimate was then exponentiated to get back to the ratio scale.

Similarly, we used a logit transformation on the AUC and Uno’s C-statistic before meta-analysis because these values are bounded between 0 and 1 and their sampling distribution becomes non-normal near those limits. The metan package in Stata 18.0 and the metafor() package in R 4.4.2 were used (Harris *et al.*, 2008, R Core Team, 2024, StataCorp, 2023, Viechtbauer, 2010).

Decision curve analysis

This technique compares the potential outcomes of using a prediction model for clinical decision making in terms of net benefit: defined as the sum of true positives minus false positives, weighted by the relative harm of false positive and false negative predictions (Vickers and Elkin, 2006). This balance is calculated for a given threshold probability, which represents the probability of the outcome at which intervention would be justified, i.e., at which the relative harms of treatment would be equal or less than the relative harms of non-intervention. In traditional prediction modelling, most of the outcomes being predicted for a given condition are negative: the occurrence of death or disease. In reproductive medicine, uniquely, we are predicting a positive outcome – live birth.

Therefore, unlike the traditional scenario where you are weighing the decision to intervene on a patient with a high predicted probability of a negative outcome, we are assessing the decision to offer ART to a patient with a low predicted probability of a positive outcome. This changes our definitions of true and false positives relative to the approach taken by Vickers and Elkin (2006), requiring some modifications to the formulae used. We have adapted the approach recommended by the STRATOS initiative (McLernon *et al.*, 2023), as detailed below.

At a given threshold probability of treatment-independent live birth:

$$Net benefit=\frac{{True positives}_{(t)}}{n}-w_{(t)}\frac{{False positives}_{(t)}}{n}$$

Where, at time *t:*

$${TP}_{\left( t \right)}= \left[ S_{\left( t, X=1 \right)} \right]*P_{\left( X=1 \right)}*N$$

True positives represent those correctly identified by the prediction model as likely to benefit from treatment – i.e. those with a low predicted probability of the event (treatment-independent live birth), who in the observed data did not go on to have a live birth (i.e. who ‘survived’).

$S_{\left( t, X=1 \right)}$ is the observed **survival** probability calculated using Kaplan-Meier (KM), and $P_{\left( X=1 \right)}$ is the probability of a positive classification (i.e. predicted chance of live birth < threshold probability).

$${FP}_{(t)}= \left[ {1-S}_{(t, X=1)} \right]*P_{\left( X=1 \right)}*N$$

False positives represent those incorrectly identified as having a low probability of treatment-independent live birth by the model – i.e. those who in the observed data **did** go on to have a live birth (i.e. the “event”). These people would have been harmed by unnecessary intervention.

${1-S}_{(t, X=1)}$ is the observed **event** probability (KM).

$$w_{(t)}= \frac{1-P_{t}}{P_{t}}$$

Where *w(t)* represents the harm-to-benefit ratio. *Pt* is the risk threshold at time *t*.

Note that in traditional DCA, the formula for these would be

$${TP}_{\left( t \right)}= \left[ 1-S_{\left( t, X=1 \right)} \right]*P_{\left( X=1 \right)}*N$$

$${FP}_{(t)}= \left[ S_{(t, X=1)} \right]*P_{\left( X=1 \right)}*N$$

$$w_{(t)}= \frac{P_{t}}{1-P_{t}}$$

As traditionally, a true positive would represent a patient who was predicted to be at high risk of an event (i.e. disease) who in the observed data did indeed have the event rather than a patient who survived event-free: therefore, they have benefited from correct intervention. Meanwhile a false positive would represent a patient that unexpectedly survived event-free despite being predicted at high risk of the event and has been treated unnecessarily.

The harm-to-benefit ratio is inverted in our formula, to mathematically reflect the difference in the definitions of true/false positives and negatives when the aim is to treat those with a predicted risk of event below the given threshold probability.

**References**

Harris RJ, Deeks JJ, Altman DG, Bradburn MJ, Harbord RM, Sterne JAC. Metan: Fixed- and Random-Effects Meta-Analysis. *Stata J* 2008;**8**:3–28.

McLernon DJ, Giardiello D, Van Calster B, Wynants L, van Geloven N, van Smeden M, Therneau T, Steyerberg EW, Bossuyt P, Boyles T *et al.* Assessing Performance and Clinical Usefulness in Prediction Models With Survival Outcomes: Practical Guidance for Cox Proportional Hazards Models. *Ann Intern Med* 2023;**176**:105–114.

R Core Team. R: A Language and Environment for Statistical Computing. 2024;**4.4.2**.

StataCorp. Stata Statistical Software: Release 18. 2023;**18**.

Vickers AJ, Elkin EB. Decision Curve Analysis: A Novel Method
for Evaluating Prediction Models. *Med Decis Making* 2006;**26**:565–574.

Viechtbauer W. Conducting meta-analyses in R with the metafor package. *J Stat Softw* 2010;**36**:1–48.

Supplementary File S3

Sensitivity analyses

Inverse probability of censoring weighting

The ipwtm() function from the *ipw* package in R was used to generate stabilised time-dependent inverse probability of censoring weights for every couple, using the approach described by van Geloven *et al.* (van der Wal and Geskus, 2011, van Geloven *et al.*, 2014). Three separate IPCW weights were generated for each of the three treatment-censoring points (OI, IUI and IVF) using the ipwtm() function. The same predictors used in the final model were used in the IPCW model formulae, as the covariates that predict chance of natural conception are all clinically relevant to the decision to start one treatment or another. The three weights were then multiplied together to generate a single combined weight, which was incorporated into the final Cox proportional hazards model to predict chance of treatment-independent live birth.

The first imputed dataset only was used for illustrative purposes.

**Supplementary File 3 Table 1:** Effect and importance (in descending order) of each couple characteristic on the chance of live birth from natural conception in the first year from diagnosis, leading to live birth. Model weighted by inverse probability of censoring, fitted in the first imputation only.

| **Characteristic (female partner unless specified)** | **HR (95% CI)*^1^*** | **p-value** |
| --- | --- | --- |
| Age of female partner (years) (36 vs. 28)^2^ | 0.62 (0.55, 0.70) | <0.0001* |
| Duration of infertility, winsorized (years) (3 vs. 1.33)^2^ | 0.64 (0.57, 0.72) | <0.0001* |
| Female secondary infertility | 1.30 (1.12, 1.50) | <0.001* |
| Smoking history (ever) (yes vs. no) | 0.74 (0.62, 0.89) | 0.001* |
| Female BMI - winsorized (kg/m^2) (28.8 vs. 21.9)^2^ | 0.88 (0.78, 0.98) | 0.023* |
| History of alcohol use (ever) | 1.08 (0.91, 1.28) | 0.4 |
| Year of first registration (2012 vs. 2003)^2^ | 1.09 (0.96, 1.23) | 0.188 |
| **Diagnosis of infertility** |  |  |
| Tubal infertility | 0.62 (0.47, 0.81) | <0.001* |
| Male factor infertility | 0.71 (0.57, 0.90) | 0.004* |
| Other infertility | 0.68 (0.50, 0.92) | 0.01* |
| Unexplained infertility | 1.29 (1.00, 1.67) | 0.05 |
| Endometriosis | 0.83 (0.55, 1.24) | 0.4 |
| Anovulatory infertility | 1.12 (0.87, 1.43) | 0.4 |
| Likelihood ratio test = 327.505, *p* = <0.0001* Wald test = 289.6, *p* = <0.0001*, Score (logrank) test = 300.147, *p* = <0.00018 AIC:15825 vs. basic model: 15065 | | |
| ^1^HR = Hazard Ratio (adjusted), CI = Confidence Interval, AIC = Akaike Information Criterion ^2^ Variables fitted as restricted cubic splines are presented as interquartile hazard ratios to ease interpretation - i.e. the hazard of natural conception leading to live birth for the 75th percentile vs. the 25th percentile value. **^*^** Statistically significant difference at p<0.05, tested using Wald test. | | |

The inclusion of inverse probability of censoring weights did not have a major effect on model parameters (Supplementary File 3 Table 1), nor the model fit, as measured by the Akaike information criterion (AIC). The predictions generated were comparable to those from the primary model as shown in Supplementary File 3 Figure 1.

**
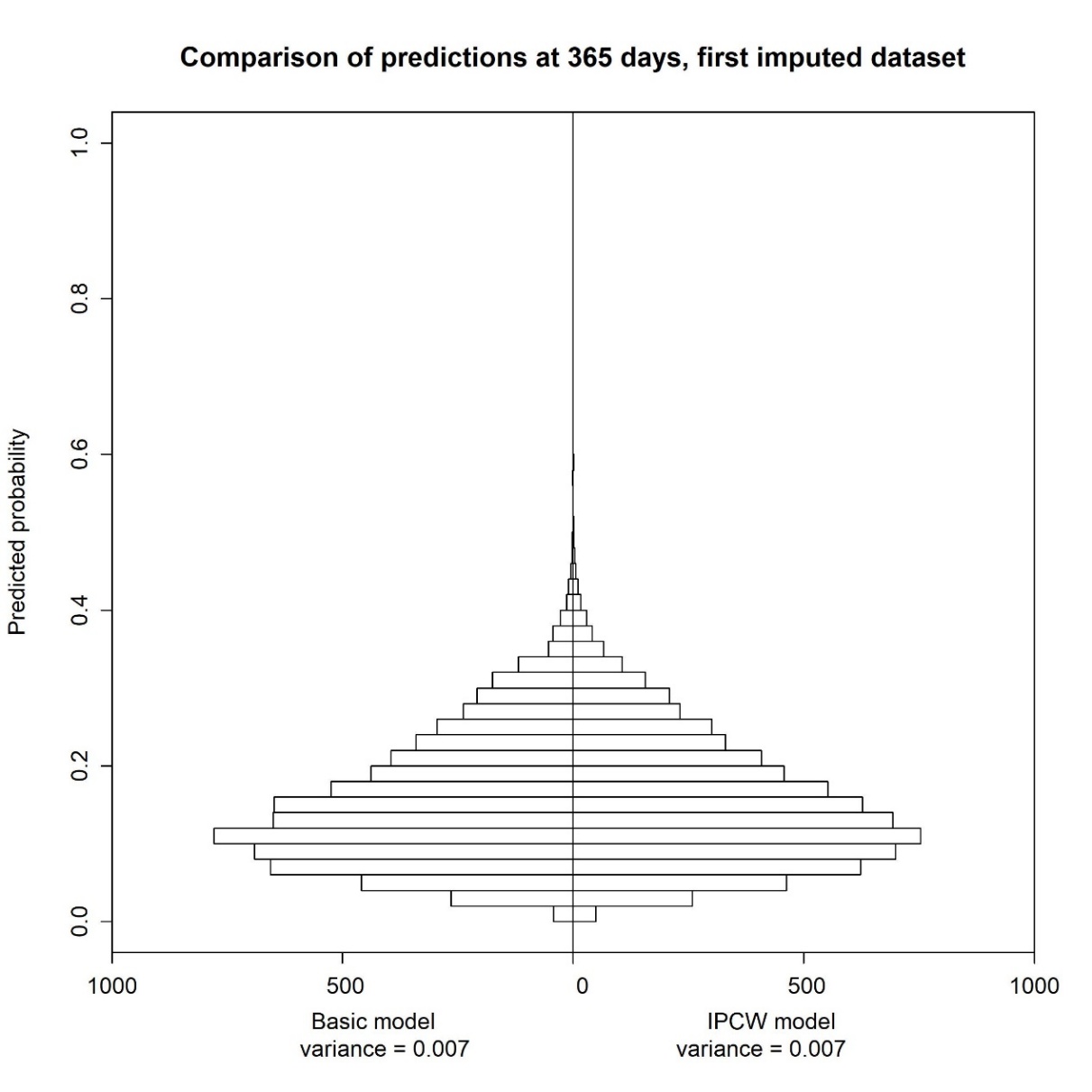
**

**Supplementary File 3 Figure 1**: Histogram comparing distribution and variance of predicted probability of live birth after natural conception at one year from diagnosis, comparing basic model and model using IPCW. Results are from first imputed dataset only for illustrative purposes. *Abbreviations:* IPCW = inverse probability of censoring weighting.

Interactions

We tested whether the inclusion of interactions terms between different diagnosis groups and female age or duration of infertility would improve model fit. We created two additional models: one including an interaction between female age (fitted as restricted cubic spline as in original model) and each diagnosis of infertility and a second with interactions between duration of infertility (again fitted as a restricted cubic spline) and each diagnosis of infertility.

Supplementary File 3 Figure 2 compared the predicted probabilities between the basic model (no interaction terms) and each of these two further models. Model fit (as calculated with AIC) was not improved by addition of these interaction terms and spread of predicted probabilities was comparable.

We also performed sample size calculations comparing the basic and interaction models using the pmsampsize() function in R (Ensor, 2023), specifying a C-statistic of 0.647 (the optimism-corrected C-statistic of the basic model). For the basic model, a minimum sample size of 6290 was required. For both models with interaction terms, a sample size of 10295 was required, which exceeded the size of our population. The decision was taken to proceed without interaction terms.

**
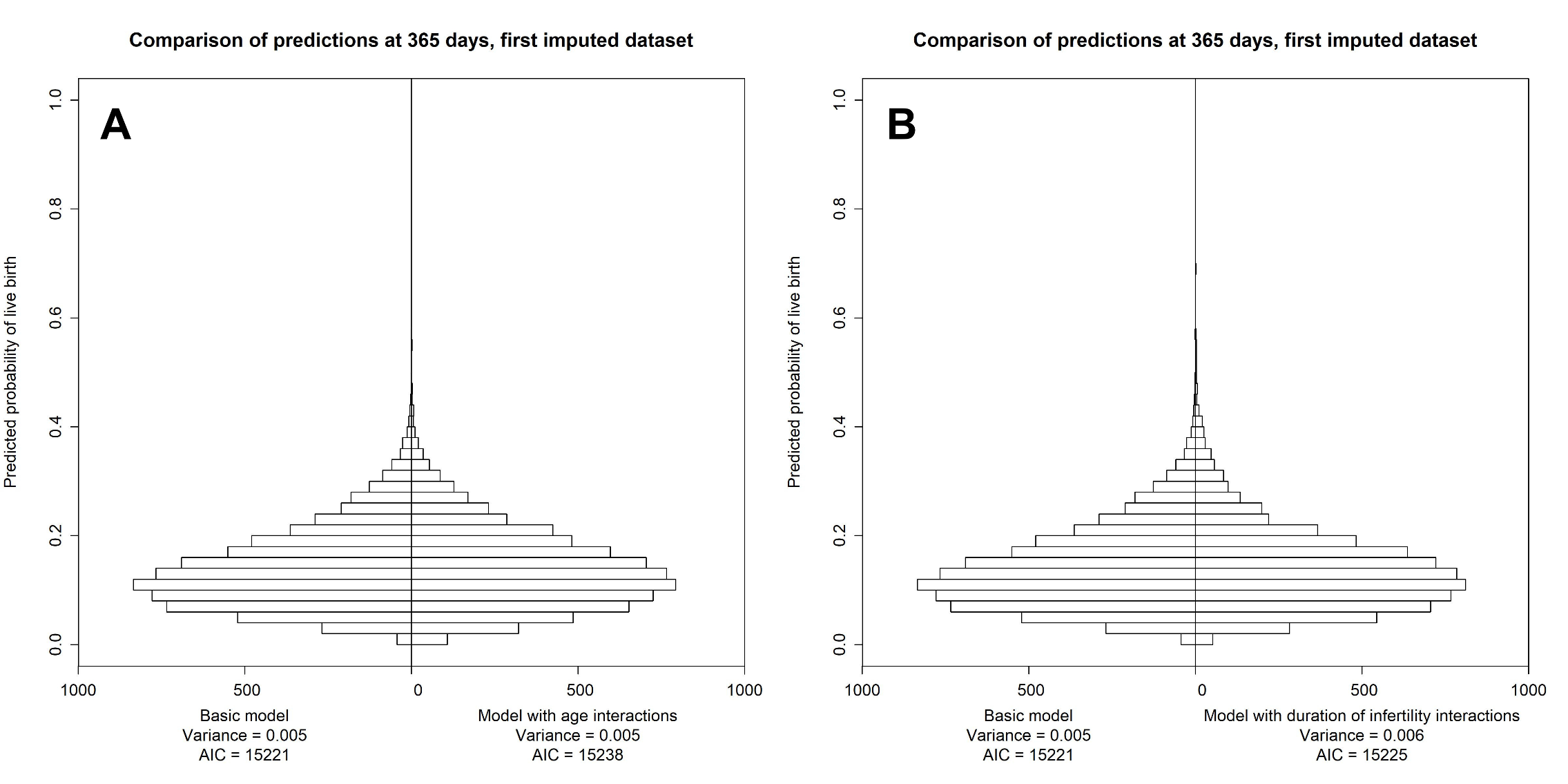
Supplementary File 3 Figure 2**: Histograms of predicted probability of live birth after natural conception at one year from diagnosis, comparing basic and models with interactions between **A:** female age and type of infertility, **B**: duration of infertility and type of infertility. Neither model showed a major change in spread of predicted probabilities, and model fit (AIC) was not improved by the addition of interaction terms. Results are from first imputed dataset only for illustrative purposes. *Abbreviations:* AIC: Akaike Information Criterion.

**References**

Ensor J. pmsampsize: Sample Size for Development of a Prediction Model. 2023;**1.1.3**.

van der Wal WM, Geskus RB. ipw : An R Package for Inverse Probability Weighting. *J Stat Softw* 2011;**43**:1–23.

van Geloven N, Geskus R , Mol B , Zwinderman A . Correcting for the dependent competing risk of treatment using inverse probability of censoring weighting and copulas in the estimation of natural conception chances. *Stat Med* 2014;**33**:4671–4680.

Supplementary File S4

# Multiple imputation

Multiple imputation by chained equations was performed using the mice() package in R (van Buuren and Groothuis-Oudshoorn, 2011). This package uses the Markov Chain Monte Carlo method to estimate the values of missing predictors based on known information. We included all baseline characteristics listed in Supplementary Table S1, along with the Nelson-Aalen estimate of cumulative hazard function (White and Royston, 2009) and key medical history variables that might inform the imputation (female smoking history (ever vs. never); female history of alcohol use (yes vs. no); female diabetes (yes vs. no), female renal disease (yes vs. no), female cardiovascular disease (yes vs. no), female respiratory disease (yes vs. no), female neurological disease (yes vs. no), female connective tissue disorder (yes vs. no), previous tubal surgery (yes vs. no), previous ovarian surgery (yes vs. no), previous sterilisation (yes vs. no), cervical disease (yes vs. no), height of male partner (cm), weight of male partner (kg); age of male partner; male smoking history (ever vs. never); history of alcohol use in male partner (yes vs. no)).

Instead of imputing BMI directly, both male and female weight and height were included in the imputation and for cases where BMI was missing, it was calculated from these imputed values.

40 imputed datasets were generated, and models were fitted across all imputed datasets and coefficients were then pooled to generate a single model representative of all imputed datasets.

**References**

van Buuren S, Groothuis-Oudshoorn K. mice : Multivariate Imputation by Chained Equations in R. *J Stat Softw* 2011;**45**:1–67.

White IR, Royston P. Imputing missing covariate values for the Cox model. *Stat Med* 2009;**28**:1982–1998.

Supplementary File S5

Calculating the risk of treatment-independent live birth

The chance of LB at 365 days can be calculated as:

$$S_{365}={0.878}^{\exp\left( PI \right)}$$

Where 0.878 is the baseline risk at 365 days, and PI is the prognostic index (calculated from patient characteristics, as below). To address overfitting, when making predictions for women with endometriosis multiply the PI by 0.59 (shrinkage factor derived from calibration slope) and for couples with “Other” infertility, it should be multiplied by 0.60.

Prognostic index formula:

$$PI=\left( 0.046 \times\left( rcsFirstYr-4.748 \right) \right) +\left( 0.007 \times\left( female age-32.258 \right) \right)-\left( 0.088 \times\left( rcsFAge-3.885 \right) \right) -\left( 0.356 \times\left( duration of infertility-2.439 \right) \right)+\left( 0.372 \times\left( rcsduryrs-0.464 \right) \right)+\left( 0.020 \times\left( rcsBMI-2.260 \right) \right)+0.243\times0.559 \left( if previous pregnancy history in female partner \right) OR +0.243\times-0.441 \left( if NO previous pregnancy history in female partner \right) -0.269\times0.775 \left( if history of smoking in female partner \right) OR -0.269\times-0.225 \left( if NO history of smoking in female partner \right)+ 0.054\times0.249 \left( if history of alcohol use in female partner \right) OR + 0.054\times- 0.751 \left( if NO history of alcohol use in female partner \right)-0.315 \times0.693 \left( if diagnosis of male factor infertility \right) OR-0.315 \times-0.307 \left( if NO diagnosis of male factor infertility \right)-0.223 \times0.954 \left( if diagnosis of endometriosis \right) OR-0.223 \times-0.046 \left( if NO diagnosis of endometriosis \right) -0.130 \times0.745 \left( if diagnosis anovulatory infertility \right) OR-0.130 \times-0.255 \left( if NO diagnosis anovulatory infertility \right) + 0.301 \times0.745 \left( if diagnosis of unexplained infertility \right) OR + 0.301 \times-0.255 \left( if NO diagnosis of unexplained infertility \right) -0.443 \times0.821 \left( if diagnosis of tubal infertility \right) OR-0.443 \times-0.179 \left( if NO diagnosis of tubal infertility \right)-0.339 \times0.919 \left( if diagnosis of other infertility \right) OR -0.339 \times-0.081(if NO diagnosis of other infertility)$$

where rcsFirstYr, rcsFAge, rcsduryrs and rcsBMI are terms from the restricted cubic splines for year of first registration, female age, duration of infertility and female BMI respectively.

These can be calculated as:

For year of first treatment:

$rcsFirstYr={\max\left( \frac{Year of first registration-1999}{k_{FirstYr}}, 0 \right)}^{3}-2.5 \times{\max\left( \frac{Year of first registration-2008}{k_{FirstYr}}, 0 \right)}^{3}+1.5 \times{\max\left( \frac{Year of first registration-2014}{k_{FirstYr}}, 0 \right)}^{3}$

Where *k* represents the normalisation factor, i.e. 15^2/3^.

For female age:

$rcsFAge={\max\left( \frac{Female age-25}{k_{FAge}}, 0 \right)}^{3}-2 \times{\max\left( \frac{Female age-32}{k_{FAge}}, 0 \right)}^{3}+1 \times{\max\left( \frac{Female age-39}{k_{FAge}}, 0 \right)}^{3}$

Where *k* represents the normalisation factor, i.e. 14^2/3^.

For duration of infertility:

$rcsduryrs ={\max\left( \frac{Duration of infertility-1}{k_{duryrs}}, 0 \right)}^{3}-1.33 \times{\max\left( \frac{Duration of infertility-2}{k_{duryrs}}, 0 \right)}^{3}+0.33 \times{\max\left( \frac{Duration of infertility-5}{k_{duryrs}}, 0 \right)}^{3}$

Where *k* represents the normalisation factor, i.e. 4^2/3^.

For female BMI:

$rcsBMI ={\max\left( \frac{Female BMI - 20.08}{k_{BMI}}, 0 \right)}^{3}-1.47 \times{\max\left( \frac{Female BMI-24.61}{k_{BMI}}, 0 \right)}^{3}+0.47 \times{\max\left( \frac{Female BMI-34.29}{k_{BMI}}, 0 \right)}^{3}$

Where *k* represents the normalisation factor, i.e. 14.21^2/3^.
