# Supplementary material for: Predicting natural conception leading to live birth for couples with infertility: a single-centre population-based cohort study of 7086 couples
Source: Hum Reprod Open. 2026 Jun 13;2026(3):hoag056. doi: 10.1093/hropen/hoag056 (PMC13353215; doi:10.1093/hropen/hoag056)
Supplement: hoag056_Supplementary_Data [file hoag056_supplementary_data.zip › Supplementary_Table_S4.docx]

**Supplementary Table S4:** Performance measures assessing the accuracy and reliability of the predictions generated by the model in the dataset used for development (apparent validation) and in the underlying population (internal validation), by subgroup of type of infertility^1^.

| **Diagnosis** | **Apparent validation (95% CI)** | **Internal validation: optimism-corrected performance (95% CI)** |
| --- | --- | --- |
| **Mean calibration (O/E)** | | |
| Other^2^ | 1.016 (0.972, 1.061) | 1.036 (0.989, 1.084) |
| Tubal | 1.029 (1.003, 1.057) | 1.050 (1.022, 1.079) |
| Unexplained | 0.979 (0.966, 0.992) | 0.983 (0.970, 0.996) |
| Endometriosis | 1.073 (1.018, 1.131) | 1.122 (1.060, 1.188) |
| Ovulatory | 0.995 (0.979, 1.011) | 0.996 (0.980, 1.013) |
| Male factor | 0.998 (0.977, 1.020) | 1.001 (0.979, 1.022) |
| **Calibration slope** | | |
| Other^2^ | 0.686 (0.620, 0.751) | 0.601 (0.534, 0.668) |
| Tubal | 1.082 (1.027, 1.137) | 1.007 (0.950, 1.064) |
| Unexplained | 1.004 (0.963, 1.045) | 0.961 (0.922, 1.000) |
| Endometriosis | 0.685 (0.568, 0.801) | 0.592 (0.476, 0.708) |
| Ovulatory | 0.966 (0.926, 1.007) | 0.913 (0.874, 0.952) |
| Male factor | 1.089 (1.046, 1.131) | 1.019 (0.978, 1.061) |
| **Uno C-statistic** | | |
| Other^2^ | 0.606 (0.595, 0.616) | 0.593 (0.582, 0.604) |
| Tubal | 0.657 (0.650, 0.665) | 0.648 (0.640, 0.656) |
| Unexplained | 0.616 (0.611, 0.621) | 0.611 (0.606, 0.616) |
| Endometriosis | 0.603 (0.585, 0.620) | 0.593 (0.576, 0.610) |
| Ovulatory | 0.631 (0.626, 0.636) | 0.625 (0.619, 0.630) |
| Male factor | 0.637 (0.631, 0.642) | 0.628 (0.623, 0.633) |
| **AUC (at 365 days)** | | |
| Other^2^ | 0.612 (0.599, 0.624) | 0.599 (0.586, 0.611) |
| Tubal | 0.682 (0.674, 0.691) | 0.672 (0.664, 0.680) |
| Unexplained | 0.624 (0.618, 0.630) | 0.618 (0.612, 0.624) |
| Endometriosis | 0.601 (0.581, 0.621) | 0.591 (0.571, 0.610) |
| Ovulatory | 0.646 (0.640, 0.652) | 0.639 (0.633, 0.645) |
| Male factor | 0.652 (0.646, 0.658) | 0.643 (0.637, 0.649) |
| ^1^ All performance measures from the model developed using the 40 imputed datasets were pooled using a random-effects inverse-variance model (Supplementary File S2).  *Abbreviations:* CI = confidence interval; O/E = observed/expected; AUC = Area under the curve.  ^2^ Other infertility is inclusive of uterine malformation, cervical factor and sexual dysfunction. | | |
