# Supplementary material for: Predicting natural conception leading to live birth for couples with infertility: a single-centre population-based cohort study of 7086 couples
Source: Hum Reprod Open. 2026 Jun 13;2026(3):hoag056. doi: 10.1093/hropen/hoag056 (PMC13353215; doi:10.1093/hropen/hoag056)
Supplement: hoag056_Supplementary_Data [file hoag056_supplementary_data.zip › Supplementary_Table_S2.docx]

**Supplementary Table S2:** Proportional hazards testing showed evidence of non-proportional hazards^†^.

| **Characteristic** | **Chi - squared** | **Degrees of freedom** | ***p*-value** |
| --- | --- | --- | --- |
| Year of first registration | 3.051 | 2 | 0.218 |
| Age of female partner | 0.050 | 2 | 0.975 |
| Duration of infertility, winsorized (years) | 7.539 | 2 | 0.023* |
| Female BMI - winsorized (kg/m^2) | 1.038 | 2 | 0.595 |
| History of previous pregnancy in female partner | 0.589 | 1 | 0.443 |
| Smoking history | 0.947 | 1 | 0.330 |
| Alcohol use | 0.110 | 1 | 0.740 |
| Male factor infertility | 1.334 | 1 | 0.248 |
| Endometriosis | 9.390 | 1 | 0.002* |
| Anovulation | 5.033 | 1 | 0.025* |
| Unexplained infertility | 20.589 | 1 | <0.001* |
| Tubal infertility | 12.894 | 1 | <0.001* |
| Other infertility | 3.381 | 1 | 0.066* |
| **Global** | **49.800** | **17** | **<0.001*** |
| **^*^** Statistically significant difference at p<0.05, tested using Schoenfeld residual score test. | | | |
| **^†^** However, given the short timeframe of follow-up with a single timepoint for prediction, at which the data were administratively censored, it was considered acceptable to proceed with direct modelling (van Houwelingen and Putter, 2015). *Abbreviations:* BMI = body mass index. | | | |

**References**

van Houwelingen HC, Putter H. Comparison of stopped Cox regression with direct methods such as pseudo-values and binomial regression. *Lifetime Data Anal* 2015;**21**:180–196.
