# Supplementary material for: Predicting natural conception leading to live birth for couples with infertility: a single-centre population-based cohort study of 7086 couples
Source: Hum Reprod Open. 2026 Jun 13;2026(3):hoag056. doi: 10.1093/hropen/hoag056 (PMC13353215; doi:10.1093/hropen/hoag056)
Supplement: hoag056_Supplementary_Data [file hoag056_supplementary_data.zip › Supplementary_Figure_S2.docx]

**
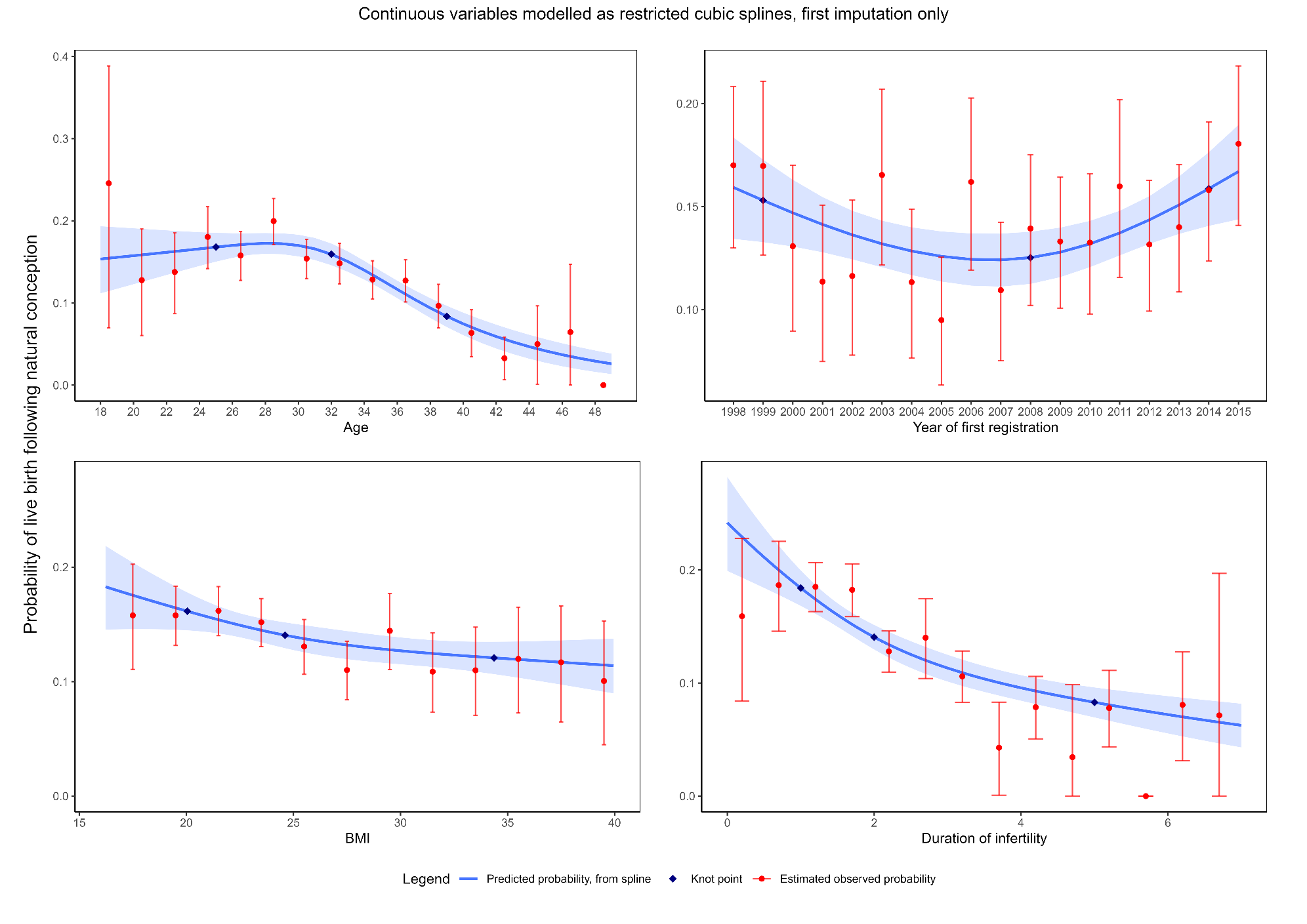
Supplementary Figure S2:** Non-linear relationships between continuous variables and the outcome of live birth following natural conception. These variables were fitted as restricted cubic splines (shown in blue) to reflect the observed relationship with the outcome (red). *Abbreviations:* BMI: body mass index.
