# Supplementary material for: Predicting natural conception leading to live birth for couples with infertility: a single-centre population-based cohort study of 7086 couples
Source: Hum Reprod Open. 2026 Jun 13;2026(3):hoag056. doi: 10.1093/hropen/hoag056 (PMC13353215; doi:10.1093/hropen/hoag056)
Supplement: hoag056_Supplementary_Data [file hoag056_supplementary_data.zip › Supplementary_Figure_S3.docx]

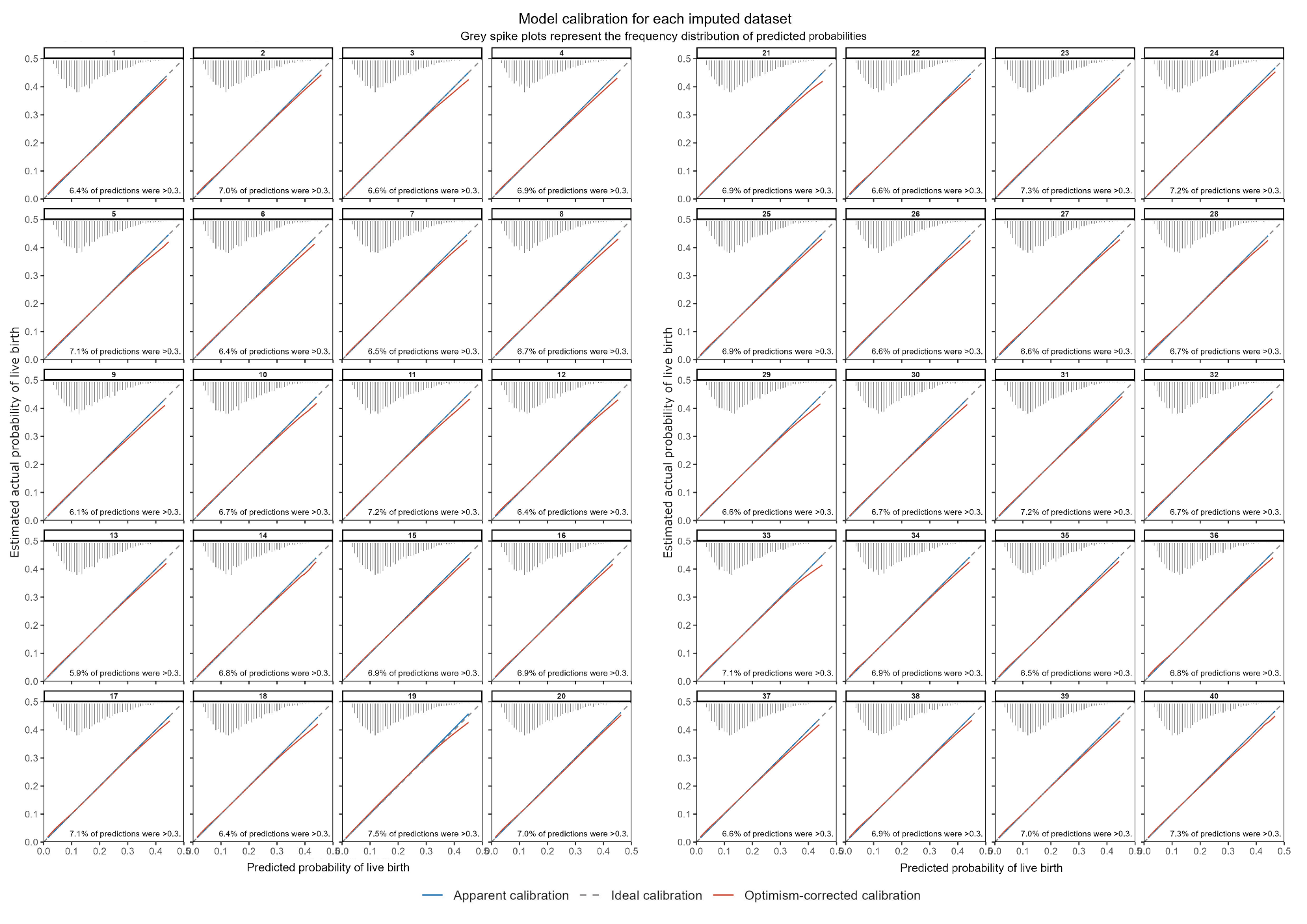


**Supplementary Figure S3:** Optimism-corrected calibration plots from each of the 40 imputed datasets, showing correlation between the model's predictions and the estimated actual observed probability of live birth following natural conception. Number above each plot represents imputation number.
