# Supplementary figures and images for: Predicting natural conception leading to live birth for couples with infertility: a single-centre population-based cohort study of 7086 couples
Source: Hum Reprod Open. 2026 Jun 13;2026(3):hoag056. doi: 10.1093/hropen/hoag056 (PMC13353215; doi:10.1093/hropen/hoag056)

**
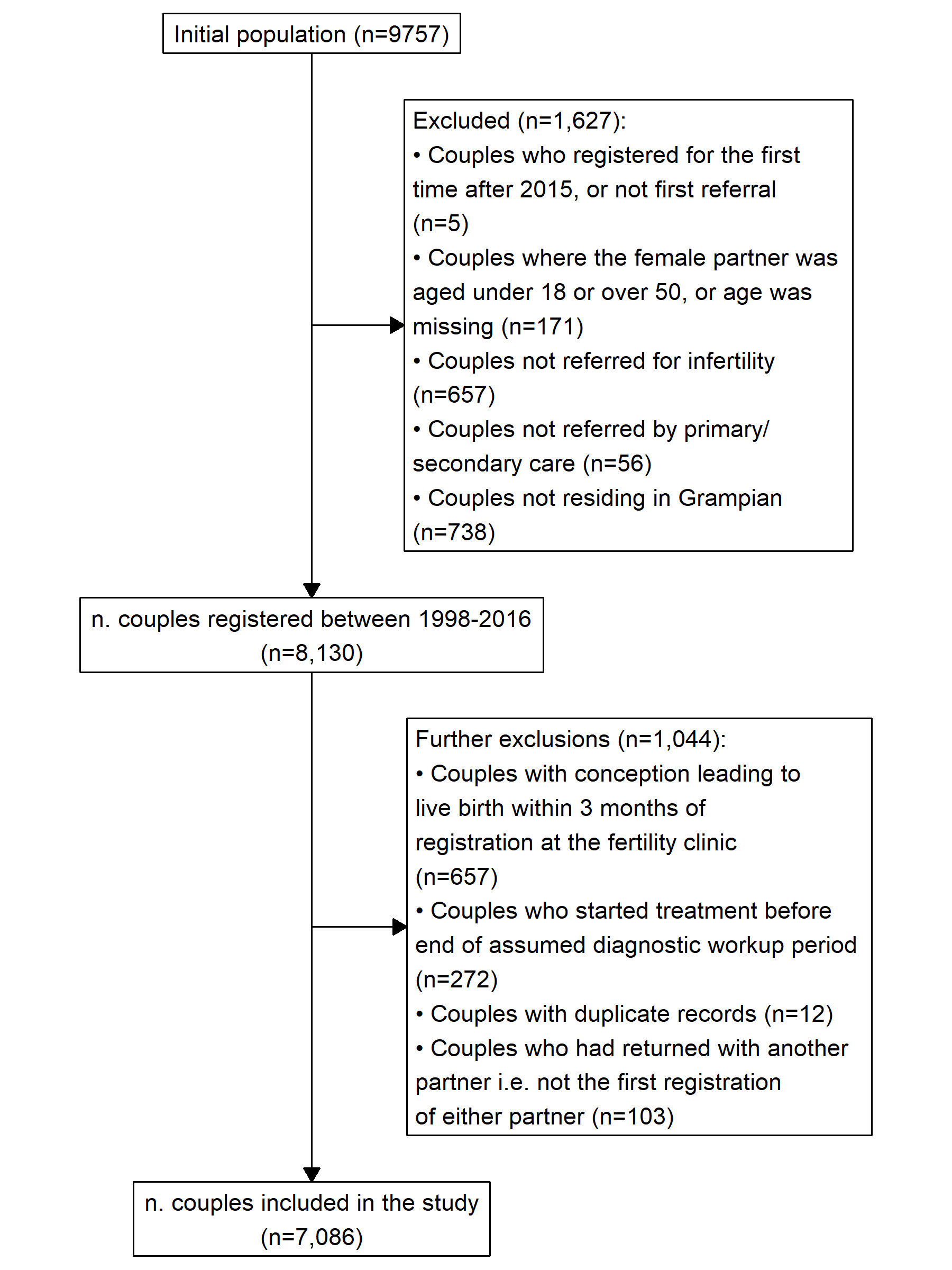
**

**Supplementary Figure S1**: Flow diagram of exclusion criteria.

Supplement: hoag056_Supplementary_Data [file hoag056_supplementary_data.zip › Supplementary_Figure_S1.docx]
